# Telomeric RNA Expression: Length Matters

**DOI:** 10.3389/fonc.2013.00178

**Published:** 2013-07-08

**Authors:** Amandine Van Beneden, Nausica Arnoult, Anabelle Decottignies

**Affiliations:** ^1^Genetic and Epigenetic Alterations of Genomes, de Duve Institute, Faculty of Pharmacy and Biomedical Sciences, Catholic University of LouvainBrussels, Belgium

**Figure 1:**
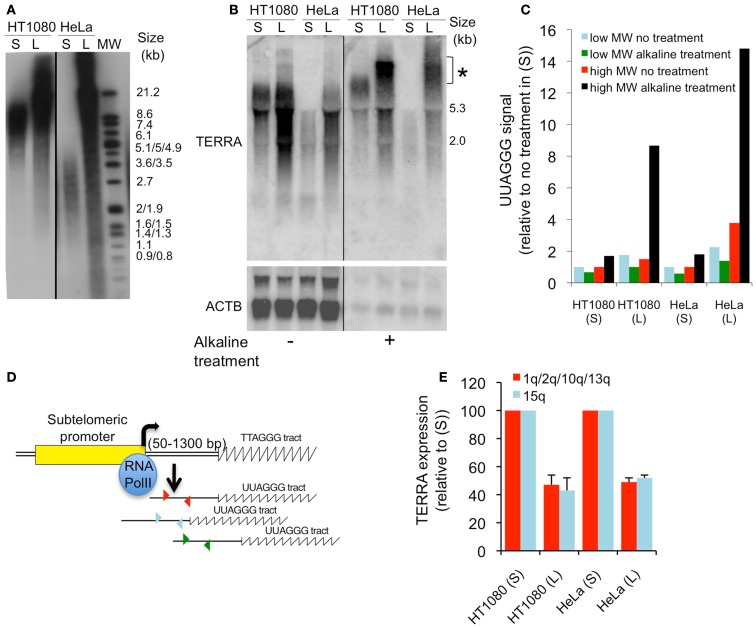
**(A)** Southern blot analysis of telomere length in HT1080 parental (S) or telomerase-overexpressing (L) cells and in HeLa parental (S) or a subclone with long telomeres (L) (2). MW, molecular weight. **(B)** Northern blot analysis of TERRA and ACTB (control) in the four cell lines described in **(A)**, with or without alkaline treatment of the agarose gel prior to transfer. *High molecular weight molecules that only transfer after alkaline treatment. **(C)** Quantification from **(B)**. **(D)** Cartoon representing human chromosome ends and subtelomeric primers (arrowheads) that are used to quantify TERRA molecules from various chromosome ends by qRT-PCR. **(E)** qRT-PCR analyses of TERRA molecules transcribed from either 1q/2q/10q/13q or 15q in the four cell lines described in **(A)**. Values were normalized to parental cell lines and error bars represent SD. All methods used here have been described in Arnoult et al. (2).

We read with great interest the Research Article by Smirnova et al. (1) in which sets of isogenic subclones isolated from human cancer cell lines were used to investigate the impact of telomere length on telomeric repeat-containing RNA (TERRA) expression. The first conclusion from this study, i.e., that telomere length does not impact on TERRA expression, was based on results obtained from a combination of Southern (telomeres) and northern (TERRA) blots. Using the same ^32^P-α[dCTP]-labeled telomeric probe, the authors, on one hand, measured telomere length by evaluating the molecular weight range in which TTAGGG signals were detected, and, on the other hand, measured TERRA expression levels by quantifying radioactive signals coming from hybridization of the probe with UUAGGG repeats. We believe, however, that the latter approach is misleading when used to quantify TERRA expression in cells with distinct telomere lengths for two main reasons. First, TERRA transcript length is naturally increased upon elongation of its template DNA, the telomeric repeats [(2); Figures 1A,B], and therefore northern blots measure UUAGGG content rather than the number of TERRA molecules. Second, the transfer efficiency of TERRA onto nylon membranes is drastically reduced upon increase in molecule length. Hence, if alkaline treatment is omitted after electrophoresis, long TERRA molecules simply do not transfer (Figures 1B,C). A drawback of alkaline treatment however explaining why this is usually not applied for northern blots is that it releases RNA from the gel (Figures 1B,C), making quantifications rather difficult. Smirnova et al. (1) do not appear to have included an alkaline treatment step as, despite high differences in telomere length, notably between HeLa parental cells (7 kb) and cells overexpressing telomerase (50 kb), TERRA profiles were similar (Figure 1 of their article). Most probably, a dot-blot analysis of total RNA would have revealed much higher levels of UUAGGG repeats in HeLa cells overexpressing telomerase. However, here too, data must be carefully interpreted as, despite higher UUAGGG repeat content, the number of TERRA molecules may still be lower compared to the isogenic cell line with shorter telomeres. This is easily explained as follows: if telomere length increases from 7 to 50 kb, and provided that UUAGGG tract length mirrors that of the TTAGGG tract, UUAGGG repeats may be up to sevenfold more abundant in cells with longer telomeres. If, as we reported previously (2), increased telomere length down-regulates telomere transcription, UUAGGG repeats may still be more abundant if the fold-reduction in telomere transcription does not exceed the fold-increase in telomere length. Following this argumentation, in the HeLa cell example given above, with a twofold reduction in the number of TERRA molecules transcribed from longer telomeres and no change in TERRA stability, the resulting number of UUAGGG repeats would be 3.5-fold higher, although molecule abundance would be decreased by twofold.

We briefly mentioned RNA dot-blot as another tool to monitor total cellular amounts of UUAGGG repeats. This, again, does not allow quantification of TERRA molecule abundance and should only be used to compare TERRA expression levels across cell lines if their respective telomere lengths are properly evaluated. TERRA-FISH (Fluorescent *In Situ* Hybridization), first described by Azzalin et al. (3), represents another analytical tool to monitor TERRA abundance at telomeres but does not allow quantification of all TERRA species as only part of them are located at telomeres (4). Hence, quantification of TERRA by RT-PCR (Reverse Transcriptase-PCR) targeting subtelomeric parts of the RNA molecules, as initiated by Azzalin et al. (3), appears to be more appropriate to assess TERRA expression independently of molecule length (Figures 1D,E). In this view, and fitting with our proposal that telomere transcription decreases upon elongation, close examination of Figure 3 data from Smirnova et al. (1) revealed that there might be an inverse correlation between telomere length and the number of TERRA molecules measured by quantitative RT-PCR in various HeLa subclones. However, because the cellular systems used in Smirnova et al. (1) are different from the ones described in Arnoult et al. (2), we cannot exclude that differences may exist between these two studies regarding telomere length-dependent repression of TERRA.

Altogether, it is clear that the tools currently available to monitor TERRA are not equivalent in terms of qualitative/quantitative information they provide and researchers should bear this in mind to properly interpret their data. Besides, one should be aware that northern blot experiments require to account for the various transfer efficiencies of short vs long TERRA molecules. In the future, it would be interesting to design additional subtelomeric primers for qRT-PCR measurement of TERRA molecules produced from single telomeres and in various species, as we believe that this represents a method of choice for quantification of TERRA expression. One should keep in mind however that qRT-PCR-based measurements of TERRA are also likely to detect very short molecules that may arise from incomplete transcription or other processes, such as TERRA degradation.
